# Supplementary material for: Recovery of Recombinant Avian Paramyxovirus Type-3 Strain Wisconsin by Reverse Genetics and Its Evaluation as a Vaccine Vector for Chickens
Source: Viruses. 2021 Feb 19;13(2):316. doi: 10.3390/v13020316 (PMC7922763; doi:10.3390/v13020316)
Supplement: Supplementary file 1 [file viruses-13-00316-s001.pdf]

Supplementary material

**APMV-1 (avirulent)**	111	G-G-**R**-Q-G-**R**-L 117
**APMV-3 (wisconsin)**	96	P-**R**-P-S-G-**R**-L 102
**APMV-3 (Netherlands)**	101	A-**R**-P-**R**-G-**R**-L 107
**APMV-1 (virulent)**	111	G-**R**-**R**-Q-**K**-**R**-F 117

Figure S1: F-Prot. cleavage site motif in APMVs: Basic amino acids (R=arginine, K=lysine) are in bold, mentioned numbers indicate amino acids position in the F protein [17].

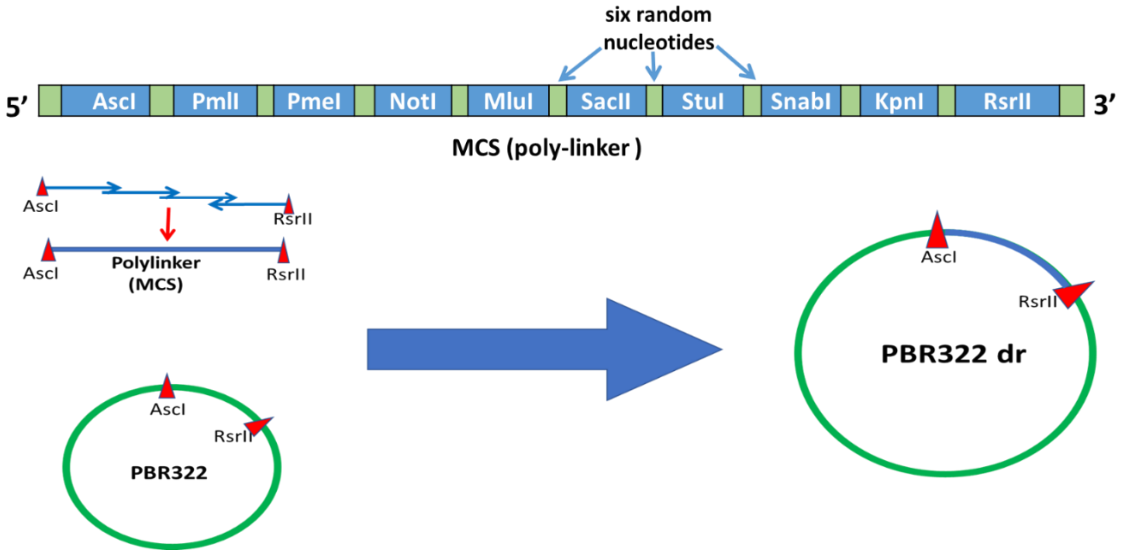

Figure S2: Preparation of the cloning plasmid vector PBR322/dr with the polylinker: A 116 nucleotides poly-linker was designed to have the same restriction enzyme sites order used to fragment the whole genome of APM3 strain Wisconsin (restriction enzyme sites were separated by random six-nucleotides). The poly-linker was created by conventional PCR using three forward and one reverse overlapping primers. The resulting gene fragment was cloned into the modified plasmid vector PBR322/dr.

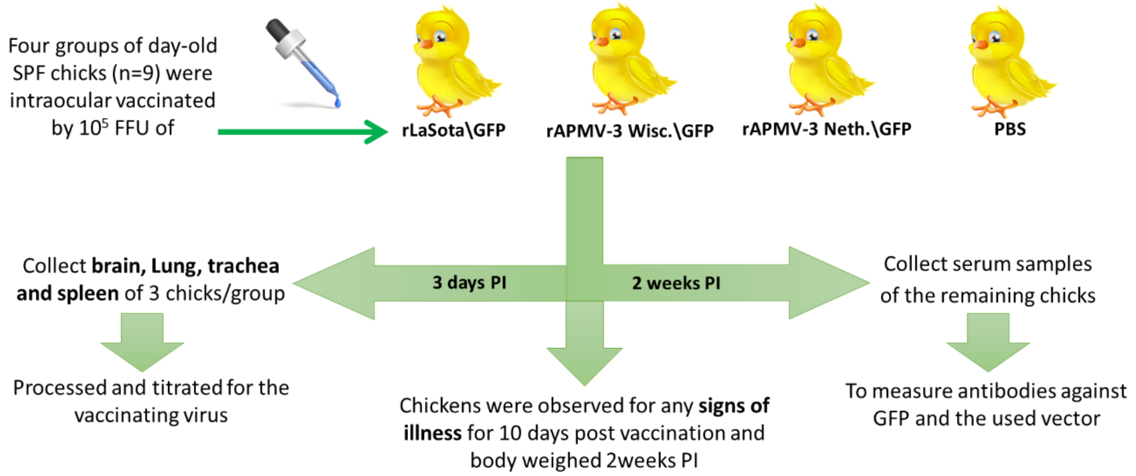

Figure S3: Schematic diagram of the in-vivo experiment for evaluation of rAPMV-3 Wisc.\GFP, rAPMV-3 Neth.\GFP and rLasota\GFP as vaccine vectors in chickens.

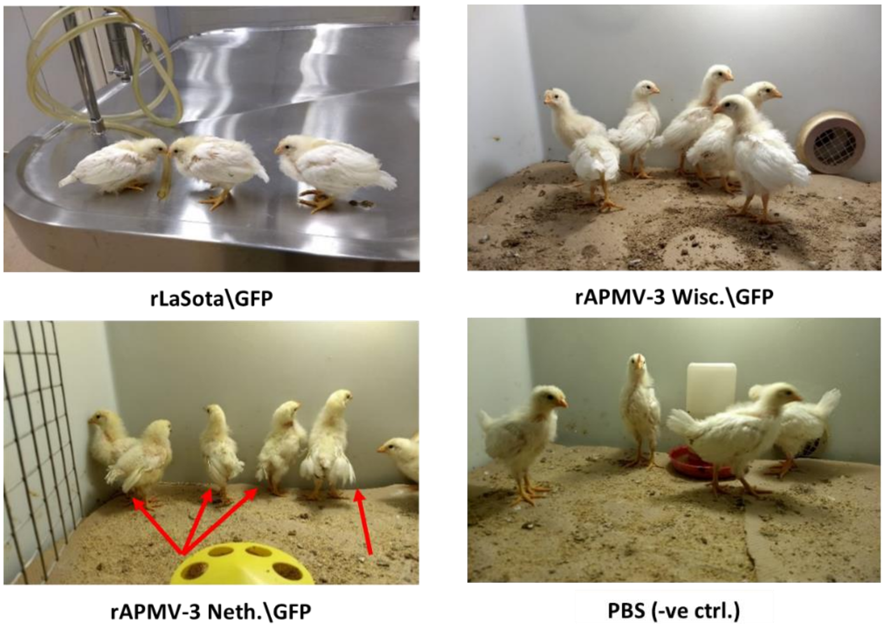

Figure S4: Pathological changes observed in vaccinated chicks (two weeks post immunization): Chicks vaccinated by rAPMV-3 Neth.\GFP are showing ruffled-abnormal feathering and stunted growth. Chicks vaccinated by rLaSota\GFP and rAPMV-3 Wisc.\GFP are showing normal body size with no signs of illness. Negative control group was mock vaccinated by PBS .

Table S1: Primers used for creation of cDNA sub-genomic fragments

Subgenomic Fragment	Primer Direction	Primers (5′-3′)	Fragment size (Kbp)
**I**	Forward<br>Reverse	ATTCGGCGCGCCTAATACGACTCACTATAGGGactaaacagaaagttaataagtgtttg<br>gtctgcAcGtgggattgggtgctgatgg	1.6 Kbp
**II**	Forward<br>Reverse	caatccccaCgTgcagacaggccaccgcctcc<br>gcaatagTtTaaaCaagagagctagatgggttgtg	1.5 Kbp
**III**	Forward<br>Reverse	tctcttGtttAaActattgctttataaaaaacc<br>ggtaagGCggcCgcagtgagtgatttatttcctg	1.4 Kbp
**IV**	Forward<br>Reverse	ctcactgcGgccGCcttaccactagtaacaaattac<br>gggtgtacGCgtgtgtctcaacatgggactgc	2.1 Kbp
**V**	Forward<br>Reverse	agacacacGCgtacaccctagtttctagtaaaacc<br>ttatctCcgCggcaaaggtgcaataatcaattc	2.1 Kbp
**VI**	Forward<br>Reverse	cctttgccGcgGagataactaagcatttaag<br>actcttaggcctgctacagctgcag	2.3 Kbp
**VII**	Forward<br>Reverse	tgtagcaggcctaagagtcgcatc<br>gcattatacgtaactatttgctc	1 Kbp
**VIII**	Forward<br>Reverse	aatagttacgtataatgcaacc<br>gattacggtaccaaactgagtgtc	2 Kbp
**IX**	Forward<br>Reverse	cagtttggtaccgtaatcgtcaatac<br>ggtcCGGACCGCGAGGAGGTGGAGATGCCATGCCGACCCactaaacaaaaagttatataaatggtttaattaac[+]	2.3 Kbp

Table S2: Nucleotide changes involved in pAPMV-3 Wisc.FLC

A

Subgenomic Fragment		Nucleotid number	Original nucleotide	Replacement nucleotide	Original sequence	New sequence	Introduced RE sites
I		Before (1)	GGCGCGCCTAATACGACTCACTATAGGG				AscI + T7 promotor
I		1590	A	C	caagcg 1588-1593	caCgTg	PmlI site
II		1592	C	T			
II		3074	A	G	attttatc 3074-3081	GtttAaAc	PmeI site
II		3078	T	A			
III		3080	T	A			
III		4391	C	G	gccgccag 4389-4396	gcGgccGC	NotI site
III		4395	A	G			
IV		4396	G	C			
IV		6517	C	G	accagt 6515-6520	acGCgt	MluI site
V		6518	A	C			
V		8577	A	G	ccacgt 8575-8580	ccGcgG	SacII site
VI	VId	8580	T	G			
VI	VId / VIc		---	---	aggcct 10896 -10901	aggcct	StuI site
VI	VIc / VIb		---	---	tacgta 11916 -11921	tacgta	SnabI site
VI	VIb / VIa		---	---	ggtacc 13925 -13930	ggtacc	KpnI site
VI	VIa	After (16182)	GGGTCGGCATGGCATCTCCACCTCCTCGCGGTCCG				HDV Ribozyme + RsrII

B

Deleted RE site	Original sequence	Corrected sequence	Changed nucleotide
**SacII**	ccgcgg 6386-6391	TcgAgg	6358
			6361
**RsrII**	cggaccg 1291-1297	cCgaTcg	1264
			1267
**RsrII**	cggaccg 2682-2688	TggCccg	2682
			2685
